# Withaferin A attenuates ovarian cancer-induced cardiac cachexia

**DOI:** 10.1371/journal.pone.0236680

**Published:** 2020-07-28

**Authors:** Natia Q. Kelm, Alex R. Straughn, Sham S. Kakar

**Affiliations:** 1 James Graham Brown Cancer Center, University of Louisville, Louisville, KY, United States of America; 2 Department of Physiology, University of Louisville, Louisville, KY, United States of America; Virginia Commonwealth University Medical Center, UNITED STATES

## Abstract

Cachexia is a common multifactorial syndrome in the advanced stages of cancer and accounts for approximately 20–30% of all cancer-related fatalities. In addition to the progressive loss of skeletal muscle mass, cancer results in impairments in cardiac function. We recently demonstrated that WFA attenuates the cachectic skeletal muscle phenotype induced by ovarian cancer. The purpose of this study was to investigate whether ovarian cancer induces cardiac cachexia, the possible pathway involved, and whether WFA attenuates cardiac cachexia. Xenografting of ovarian cancer induced cardiac cachexia, leading to the loss of normal heart functions. Treatment with WFA rescued the heart weight. Further, ovarian cancer induced systolic dysfunction and diastolic dysfunction Treatment with WFA preserved systolic function in tumor-bearing mice, but diastolic dysfunction was partially improved. In addition, WFA abrogated the ovarian cancer-induced reduction in cardiomyocyte cross-sectional area. Finally, treatment with WFA ameliorated fibrotic deposition in the hearts of tumor-bearing animals. We observed a tumor-induced MHC isoform switching from the adult MHCα to the embryonic MHCβ isoform, which was prevented by WFA treatment. Circulating Ang II level was increased significantly in the tumor-bearing, which was lowered by WFA treatment. Our results clearly demonstrated the induction of cardiac cachexia in response to ovarian tumors in female NSG mice. Further, we observed induction of proinflammatory markers through the AT_1_R pathway, which was ameliorated by WFA, in addition to amelioration of the cachectic phenotype, suggesting WFA as a potential therapeutic agent for cardiac cachexia in oncological paradigms.

## Introduction

Ovarian cancer is one of the leading causes of cancer mortality in the US because this disease is typically diagnosed in advanced stages with widespread metastases. For average risk patients, no screening tests are available for diagnosis at early stages. Therefore, very soon after diagnosis, patients experience the clinical symptoms of cachexia: involuntary body weight loss, severe muscle wasting, fatigue, and a decreased response to anticancer therapies; these symptoms lead to a reduction in quality of life and overall survival rate [1–3]. Ovarian cancer patients frequently exhibit the complex metabolic syndrome cachexia [4], which is primarily marked by a significant loss of skeletal muscle and functional muscle weakening [2, 5]. Development and prognosis of chronic heart failure are related to nutritional status [6]. The prevalence of cardiac cachexia ranges from 10% to 39%, depending on the disease state [6, 7]. The prognosis for patients with cardiac cachexia is poor, with mortality reaching up to 50% in 18 months [6]. Several cancers have been demonstrated to have a deleterious effect on the heart, but common cancer treatments, such as chemo- and/or radiotherapy, are capable of inducing a cachectic phenotype in and of themselves or exacerbating cardiac dysfunction stemming from the cancer [8, 9].

Myocardial atrophy is a common feature observed in murine models of cancer-induced cachexia, with a decrease in heart weight of up to ~20% in tumor-bearing mice compared to that of non-tumor-bearing mice [10]. However, the same study also showed that ectopic implantation of C26 colon carcinoma cells into female mice yielded a milder atrophying effect due to the cardioprotective effects of estrogen [10]. Along similar lines, post-menopausal women have an increased risk of cardiovascular disease due to the loss of endogenous estrogen production [11]. A majority of ovarian cancer patients are post-menopausal with very low levels of circulating estrogen [12]. In addition, some xenograft models of ovarian cancer resulted in the dysregulation of the estrous cycle and/or premature termination of estrous cycling, resulting in decreased levels of circulating estrogen [13–15]. Based on this information, it is possible that female mice could be utilized as a model to study cancer-induced cachexia given the proper oncological paradigm. Therefore, in our present study, we used female NSG mice as a model to examine the induction of cardiac cachexia by ovarian cancer.

The renin-angiotensin system (RAS) is known for its crucial role in maintaining a healthy cardiovascular system, as well as fluid and electrolyte balance [16]. Angiotensin II (Ang II), the principal effector of the RAS, binds to the two distinct Ang II receptors: Ang II type 1 (AT_1_R) and Ang II type 2 (AT_2_R). Ang II stimulates angiogenesis via the upregulation of vascular endothelial growth factor [16, 17], and these Ang II-induced cellular effects are mostly mediated through the specific G protein-coupled AT_1_R [18]. In patients with ovarian cancer, expression of AT_1_R correlates with tumor angiogenesis and poor clinical outcomes [16–18]. AT_2_R is highly expressed in the fetus, rapidly reduced after birth and is upregulated in response to pathologic stimuli to have cardioprotective effect, such as hypertension and/or myocardial ischemia [19, 20]. The correlation between negative clinical outcomes and alterations in Ang II signaling led us to explore this pathway in cardiac tissue in the settings of ovarian cancer.

Previously, we have established a murine model of cancer-induced skeletal muscle cachexia [21]. In this study, we explored cancer-induced cardiac atrophy (cachexia) and the therapeutic potential of Withaferin A (WFA). WFA is a steroidal lactone that is a purified extract from the plant *Withania somnifera*, also known as Ashwagandha or winter cherry. WFA is known for its anti-inflammatory properties and inhibitory effects on tumor growth, as well as the invasion and metastasis of several cancer cell lines [22–27], including the ovarian cancer cell lines: A2780, SKOV3, and CAOV3 [28–32]. In addition to its effects on cancer cells, WFA has been shown to target cancer stem cells [30]. Recent work from our group has demonstrated that WFA ameliorates the muscle weakening and myofibrillar atrophy induced by ovarian cancer [21]. However, to the best of our knowledge, no study has investigated the effect of WFA on cardiac cachexia.

In the present study, we sought to corroborate our prior findings that WFA ameliorates global body wasting induced by ovarian cancer [21]. We also show that cardiac atrophy is due to a decrease in all myofibrillar proteins, as opposed to a decrease specifically in myosin heavy chain (MHC). In addition, we investigated whether ovarian cancer induces cachectic changes in the heart, and if WFA attenuates cardiac cachexia. Our results corroborated our previous study demonstrating that WFA attenuates the gross body changes and tumor burden associated with ovarian cancer [21]. Further, we hypothesized that xenografting of ovarian cancer into female mice would induce a cachectic phenotype in cardiac muscle through AT_1_R. Ang II released from tumor induces shift in MHC isoforms from a predominantly adult α-MHC state to one that is primarily embryonic β-MHC in the tumor-bearing vehicle-treated group compared to the tumor-free vehicle-treated group and caused cardiac cachexia. Treatment with WFA would reduce Ang II levels and attenuates cardiac cachexic phenotype. Our study shows that our xenograft model of ovarian cancer induced a cachectic phenotype. Meanwhile, WFA preserved systolic function in the tumor-bearing mice and partially attenuated diastolic dysfunction.

## Materials and methods

### Cell line

The A2780 ovarian cancer cell line was maintained in Roswell Park Memorial Institute (RPMI) 1640 medium supplemented with 10% fetal bovine serum (FBS, Hyclone), 100 U/ml penicillin, and 10 μg/ml streptomycin. Cells were cultured in a humidified atmosphere of 5% CO_2_ at 37°C, and the medium was changed every 48 hours as described previously [30].

### Generation of tumors in mice

Six-week old female NOD.Cg-*Prkdc*^*scid*^ *Il2rg*^*tm1Wjl*^/SzJ (NSG, Jackson Laboratory Strain #005557) immunodeficient mice were randomly assigned to a tumor-free or tumor-bearing group (30 mice/group). Tumor-bearing groups received an intraperitoneal (i.p.) injection of 8.0 x 10^5^ low passage A2780 cells (growing in log phase) suspended in 100 μl of sterile PBS. Tumor-free control groups received i.p. injection of 100 μl of sterile PBS alone. After an initial refractory period of eight days, mice in both the tumor-free and tumor-bearing groups were further stratified into a group that received vehicle (10% dimethyl sulfoxide, 90% glycerol trioctanoate, n = 10) or one of two doses of WFA (2 mg/kg or 4 mg/kg, n = 10) via i.p. injection. Injections were performed once every three days until the culmination of the study. Mice were monitored on a daily basis for signs of discomfort using the “Pain Scoring Using Response Variables” and the “Mouse Grimace Scale” as indicated by our IACUC protocol. Based upon the two scales utilized to monitor research animal distress/discomfort, research animals would be euthanized if they achieved a body condition score of 1, physical appearance score of 3, or behavior score of 2. Non-steroidal anti-inflammatory drugs were withheld from mice as cachexia is mediated by inflammation, in accordance with our IACUC protocol. As we employed an i.p. xenograft model of cancer, we could not visualize/measure the xenografted tumor. Therefore, we were advised by our veterinary staff to adapt a weight change threshold criterion for a humane endpoint. Per our IACUC protocol, if a mouse reached a 0.25-fold change (gain or loss) in body mass, it would be euthanized. At the termination of the study, no research animals researched this threshold (all animals were under 0.15-fold change). Mice were anesthetized using isoflurane to allow central collection of blood. After the collection of blood, the mice were euthanized via vital organ removal and exsanguination while under heavy anesthesia. A secondary method of euthanasia (cervical dislocation) was performed afterwards. After euthanization, several tissues were collected, weighed, snap frozen in liquid nitrogen, and then stored at -80°C for further analysis. During the course of the study, the mice were housed in conditions with a 12-hour light–dark cycle and were given water and food *ad libitum*. The Institutional Animal Care and Use Committee (IACUC, protocol # 15405) and Institutional Biosafety Committee (IBC, protocol # 18–208) of the University of Louisville approved all experimental protocols for mice in advance.

### Echocardiography

Left ventricular (LV) systolic and diastolic function were evaluated by transthoracic echocardiography using a Vevo 3100 system and a 40 MHz linear probe (FUJIFILM VisualSonics Inc., Toronto, Ontario, Canada) and was adapted from Bauer et al [33]. Mice were anesthetized with isoflurane, maintained at an equivalent surgical depth of anesthesia (induction chamber at 5% with 1.5–2.0 L/min O_2_ flow, followed by 1.5–2.0% with 1.5–2.0 L/min O_2_ flow), and placed in the supine position. The skin over the thorax was shaved and then briefly subjected to a depilatory cream. Body temperature was maintained at 37–38°C, and heart rate was monitored using the accompanying Vevo Imaging Station. Briefly, variables that represent diastolic function (E/A ratio, isovolumetric relaxation time (IVRT), and (E/e’) were measured during the resting condition from an apical four-chamber view with conventional pulsed wave tissue Doppler. The E/A ratio was calculated from the peak velocity flow in early diastole (the E wave) to the peak velocity flow in late diastole caused by atrial contraction (the A wave) during resting conditions. An image was captured along the parasternal short axis and analyzed offline using the Vevo^®^ Lab software. Standard measures of LV structure [i.e., LV internal diameter (LVID) and LV posterior wall thickness (PWT)] and function [i.e., stroke volume (SV), cardiac output (CO), and ejection fraction (EF)] were obtained along the parasternal short axis during resting and stress conditions. In M-mode, wall thickness and chamber dimensions across the sample line were used to calculate anatomical and functional parameters. End-diastolic and end-systolic volumes were estimated from LVID at systole. Results from five cardiac cycles during expiration were averaged together and used for between-group and within-group comparisons [34]. Continuous ECG and heart rate monitoring were performed over a period of one hour while the other measurements were collected.

### Histology and morphometric heart analysis

The hearts of the mice were isolated, flash frozen in liquid nitrogen, mounted in O.C.T. embedding medium, and then sectioned using a microtome cryostat. To assess tissue morphology, 8 μm thick transverse sections were cut from the mid-ventricle of the heart. These sections were then subjected to hematoxylin and eosin (H&E) staining or Masson’s trichrome staining. Images of H&E-stained and Masson’s trichrome-stained heart sections were quantified using Fiji software (National Institutes of Health software) to measure myofiber cross-sectional area (CSA) or the degree of collagen deposition, respectively. Cardiac myofiber CSA was calculated by analyzing 100–200 fibers per heart, as described by Helms et al [35]. Collagen deposition was quantified from the Masson’s trichrome-stained tissue and was adapted from Chen et al [36].

### Imaging

Slides were mounted using Eukitt quick-hardening mounting medium (Sigma-Aldrich) and visualized at -0.4°C on a Nikon TiE 3000 inverted microscope (Nikon) equipped with a digital camera (DS-U2/L2-Ri1 digital microscope camera (Nikon) for light microscopy or DXM-1200C coded digital camera (Nikon) for fluorescent microscopy) and Nikon NIS Elements AR software (Nikon). Exposure times were consistent for each staining type. Image levels were equally adjusted using Adobe Photoshop CS6 software (Adobe) to remove nonspecific background staining. Necropsy images were captured on a handheld digital camera and stored as high-resolution JPEG files. Margins of cropped images are indicated by a solid black or white border.

### Total RNA purification and qPCR

Isolation of total RNA from tumor samples was performed using an RNeasy Mini Kit (Qiagen Catalog # 74104) according to the manufacturer’s instructions. Isolation of total RNA from cardiac muscle was performed using an RNeasy Fibrous Tissue Mini Kit (Qiagen Catalog # 74704) according to the manufacturer’s instructions. First strand cDNA was synthesized using 1 μg of purified RNA and a commercially available kit (iScript™ cDNA synthesis kit, Bio-Rad Catalog # 170–8891). Quantification of mRNA expression was performed using quantitative real-time PCR (qPCR) as described previously [21] and the SYBR Green dye method on a StepOne Plus™ system (Applied Biosystems). The gene-specific primers used are listed in Table 1.

**Table 1:** Human and mouse gene specific primer sequences.

Gene	Species	Forward	Reverse
*α-MHC*	Mus musculus	5′-GAG ATT TCT CCA ACC CAG-3′	5′-TCT GAC TTT CGG AGG TACT-3′
*β-MHC*	Mus musculus	5′-CTA CAG GCC TGG GCT TAC CT-3′	5’-GCC ACA AGC AGG AAT GAG AA-3’
Cardiac Troponin I	Mus musculus	5’-TCT GCC AAC TAC CGA GCC TAT-3’	5′- CTC TTC TGC CTC TCG TTC CAT-3’
*IFNγ*	Mus musculus	5’-GAC AAT CAG GCC ATC AGC AAC-3’	5’-CGG ATG AGC TCA TTG AAT GCT T-3’
*TNFα*	Mus musculus	5'- AGC ACA GAA AGC ATG ATC CG -3'	5'- GCC ACA AGC AGG AAT GAG AA -3'
*IL-6*	Mus musculus	5’-CCT TCT TGG GAC TGA TGC TGG-3’	5’-GCC TCC GAC TTG TGA AGT GGT-3’
*MIP-2*	Mus musculus	5’-CCA CTC TCA AGG GCG GTC AAA-3’	5’-TAC GAT CCA GGC TTC CCG GGT-3’
*AT1aR*	Mus musculus	5'- CAT TCC TGG ATG TGC TG-3'	5'- GAA CAA GAC GCA GGC TTT -3'
*AT1bR*	Mus musculus	5'- ATG AAT CTC AGA ACT CAA CAC -3'	5'- AAA CTT GAA TAT TTG GTG GGG A-3'
β-Actin	Mus musculus	5’-CAG GCA TTG CTG ACA GGA TG-3’	5’-TGC TGA TCC ACA TCT GCT GG-3’
*AGT*	Homo sapiens	5'- CTG GCC GCC GAG AAG CTA G-3'	5'- CCC CAC CAT GAT GGA CTG TA-3
*GAPDH*	Homo sapiens	5’-TGA TGA CAT CAA GAA GGT GGT-3’	5’-TCC TTG GAG GCC ATG TGG GCC-3’

### Measurement of plasma Ang II

Plasma Ang II levels were measured with an enzyme-linked immunosorbent assay (ELISA). Blood samples were collected in tubes containing ethylenediaminetetraacetic acid (EDTA) (25 mM), *o*-phenanthroline (0.44 mM), pepstatin A (0.12 mM), and *p*-hydroxymercuribenzoic acid (1 mM) and then centrifuged at 1200 × *g* for 10 min. The plasma was collected and stored at -80 °C until further processing. Ang II levels were quantified by using a commercially available ELISA kit according to the manufacturer’s instructions (Sigma-Aldrich Catalog # MAK190).

### Ethical approval

All procedures involving the use of mice were carried out in strict accordance with the standards of the National Institutes of Health guide for the care and use of laboratory animals. The Institutional Animal Care and Use Committee (IACUC, protocol # 15405) and Institutional Biosafety Committee (IBC, protocol # 18–208) of the University of Louisville approved all experimental protocols in mice in advance. No human data or tissue was used in this study.

### Graphical display and statistical analysis

For the sake of transparency and to display the entire range of data, the majority of the results are expressed as box-and-whisker plots with the box composed of the first, second, and third quartiles and the lower and upper whiskers corresponding to the minimum and maximum values, respectively. Individual data points are depicted as black circles. Image cropping is indicated by a solid black or white line around the field of view. Statistical analysis of the data was performed using one-way analysis of variance (ANOVA) followed by Tukey’s honestly significant Difference test (HSDT) post hoc analysis for comparisons between 3 or more groups with one experimental factor or two-way ANOVA followed by Tukey’s multiple comparison post hoc test for comparisons between 4 or more groups containing two factors. Statistically significant differences between groups were determined with GraphPad Prism 8.3.0 software for Mac (La Jolla, California, USA). ANOVA summaries are presented in the supplementary information. A Tukey-corrected p-value of < 0.05 was considered statistically significant, unless otherwise specified.

## Results

### WFA ameliorates gross body changes induced by ovarian cancer

Recent work from our lab demonstrated that the A2780 ovarian cancer cell line is capable of inducing a skeletal muscle cachectic phenotype in NSG mice and treatment with WFA attenuates these changes [21]. Our present study aimed to corroborate these findings and determine whether ovarian cancer induces cardiac cachexia and if so, whether WFA reverses this phenotype. We generated ovarian tumors by i.p. injection of 8 x 10^5^ A2780 ovarian cancer cells instead of the previously reported 1 x 10^6^ cells to avoid rapid growth of i.p. tumors [21]. Additionally, we changed the doses of WFA to 2 mg/kg and 4 mg/kg instead of 2 mg/kg and 6 mg/kg because 6 mg/kg WFA turned out to be toxic in our previous study [21].

**Fig 1:**
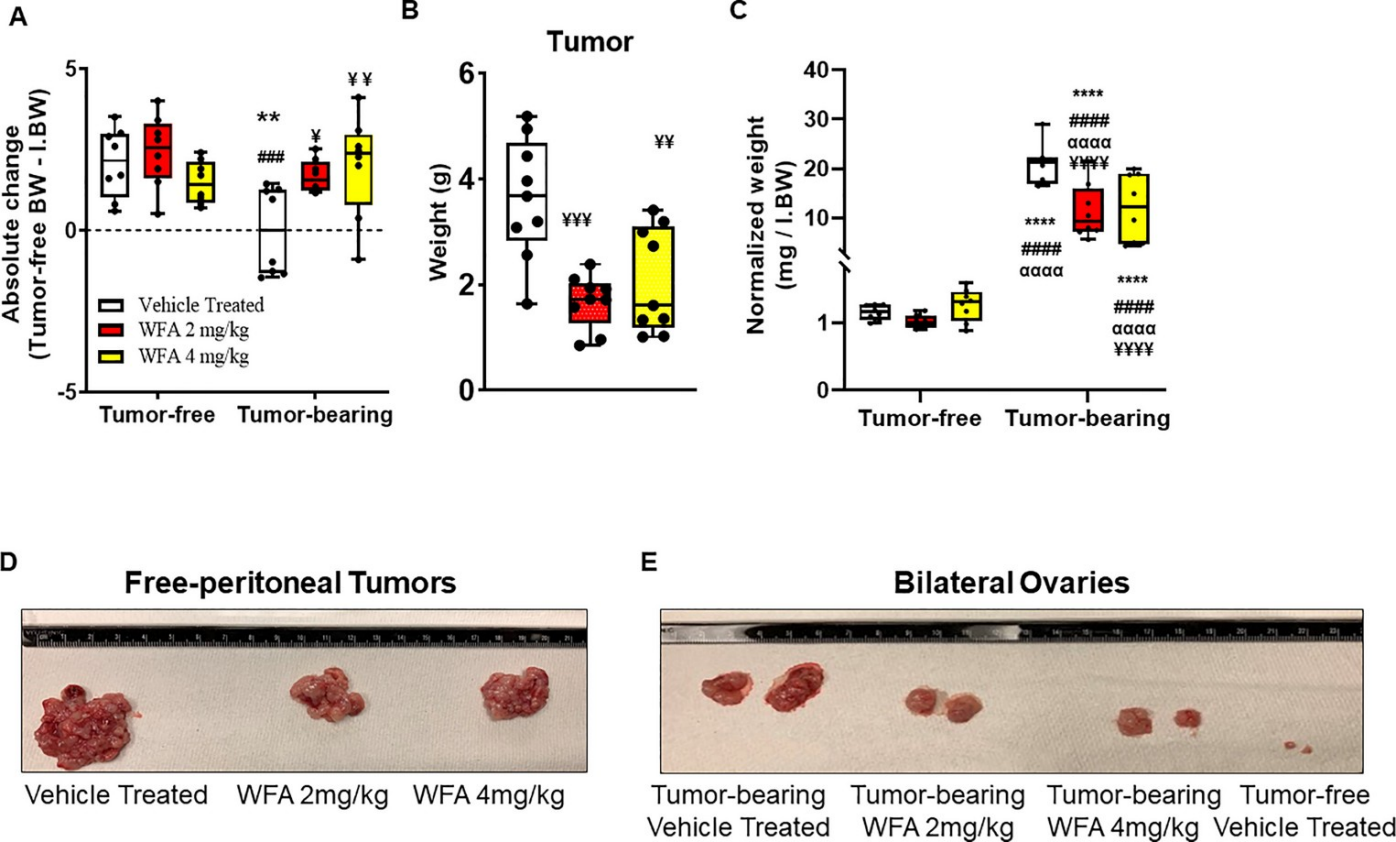
Withaferin A reduces ovarian tumor burden and inhibits body weight loss. (**A**) Absolute change in tumor-free body weight (i.e. Terminal BW–Tumor Mass–Initial BW) in tumor-free and tumor-bearing mice that were treated with WFA or vehicle. (**B**) Quantification and (**D**) representative images of free peritoneal tumor xenografts in WFA- and vehicle-treated groups. (**C**) Quantification and (**E**) representative images of the bilateral ovaries in WFA- and vehicle-treated groups. N = 8–10 per group. Black circles indicate individual data points. *p < 0.05; **p < 0.01; ***p < 0.001; or ****p < 0.0001 indicates a significant difference from the corresponding value of the tumor-free vehicle-treated group by two-way ANOVA followed by Tukey’s multiple comparison test. ^#^p < 0.05 indicates a significant difference from the corresponding value of the tumor-free WFA 2 mg/kg group. ^**α**^p < 0.05 indicates a significant difference from the corresponding value of the tumor-free WFA 4 mg/kg group. ^**¥**^p < 0.05 indicates a significant difference from corresponding the value of the tumor-bearing vehicle-treated group.

Due to the weight masking effects of ovarian cancer-associated tumor burden, to assess changes in body weight, we calculated the absolute change in tumor-free body weight (normalized to the initial body weight (IBW)) (Fig 1A). The tumor-bearing vehicle-treated group displayed an average negative change in absolute body weight (Fig 1A). The remainder of the groups displayed a positive change in absolute body weight (Fig 1A). The change in body weight of the tumor-bearing vehicle-treated group was significantly different from the tumor-free vehicle-treated group (p < 0.01) (Fig 1A). Within the tumor-bearing groups, both doses of WFA led to significant preservation of body weight loss compared to that of the tumor-bearing vehicle-treated group (WFA 2 mg/kg: p = 0.04; WFA 4 mg/kg: p = 0.009), but the differences were not significantly different from those of the tumor-free groups (p > 0.90) (Fig 1A). Consistent with our previous study [21], we found that WFA treatment significantly reduced the weight of visible tumors (WFA 2 mg/kg: 1.6 ± 0.19 g; WFA 4 mg/kg: 1.9 ± 0.35 g) compared to that of the vehicle-treated group (3.6 ± 0.43 g) (WFA 2 mg/kg: p = 0.0004; WFA 4 mg/kg: p = 0.004) (Fig 1B and 1C). No significant difference in tumor weight between the two doses of WFA was observed (p = 0.65).

We observed that i.p. tumors were metastasized to select organs/anatomic regions, such as the ovaries (Fig 1E; ovaries from the tumor-free WFA-treated groups not shown) and peri-organ fat pads (S1 Fig; only images from the vehicle-treated groups are displayed). These regions are known to be highly estrogenic responsive [37]. The normalized weights of the bilateral ovaries were found to be significantly increased in all three tumor-bearing groups compared to all three tumor-free groups, as a byproduct of tumor metastasis (21.9 ± 1.3 mg in the tumor-bearing vehicle-treated group compared to 1.13 ± 0.04 mg/g IBW in the tumor-free vehicle-treated group) (p < 0.0001 for all comparisons) (Fig 1D). However, both WFA treatments led to a significant reduction in the normalized weights of the bilateral ovaries in the tumor-bearing groups (WFA 2 mg/kg: 10.38 ± 1.32 mg/g IBW; WFA 4 mg/kg: 12.05 ± 2.09 mg/g IBW) compared to those of the tumor-bearing vehicle-treated group (WFA 2 mg/kg: p < 0.0001; WFA 4 mg/kg: p = 0.0002) (Fig 1D).

No significant differences were found in the normalized weights of other common metastatic sites of ovarian cancer, such as the brain, lungs, liver, or kidney (S1 Table). This could be attributable to differences in the metastatic model utilized versus metastatic routes that could be exhibited in a primary model of ovarian cancer. While a thorough histological examination of all peritoneal/retroperitoneal organs was not performed, we found that at a gross level, the majority of the metastatic lesions encapsulated the organs, such as the spleen (S1B Fig; only images from the vehicle-treated groups are displayed).

### Withaferin A preserves systolic function

**Fig 2:**
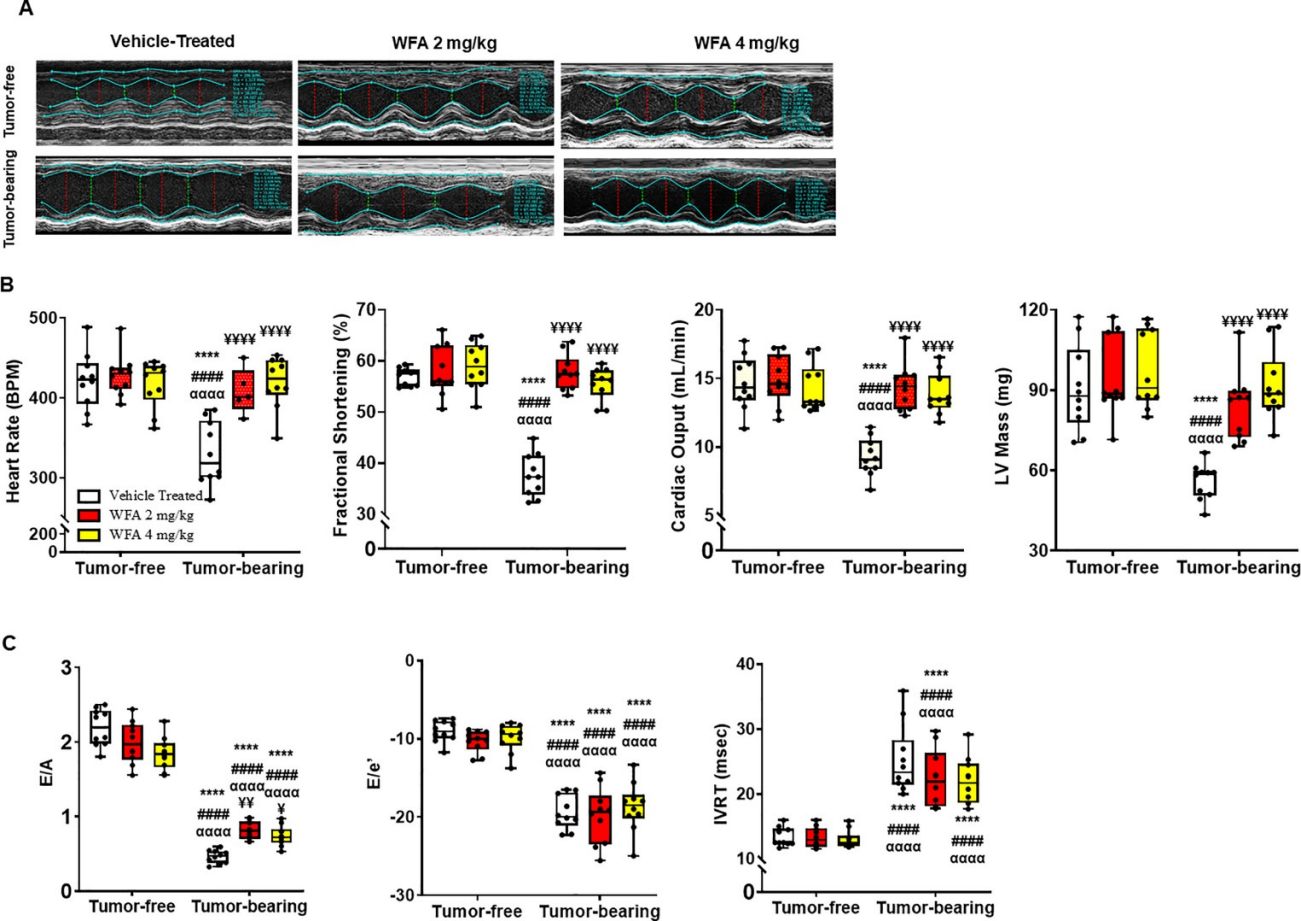
Effects of Withaferin A on left ventricular systolic and diastolic function in the context of ovarian cancer. (**A**) Representative M-mode images from all groups. (**B**) Systolic function parameters: recorded heart rate via echocardiography (BPM) in tumor-free and tumor-bearing mice that were treated with WFA or vehicle. The graphical representation of left ventricular, % fractional shortening, cardiac output, and Average calculated LV mass. Graphical representations of the (**C**) diastolic function parameters: E/A ratio, E/e’ ratio, and isovolumetric relaxation time (IVRT) in tumor-free and tumor-bearing groups that were treated with WFA or vehicle. N = 10 per group. Black circles indicate individual data points. *p < 0.05; **p < 0.01; ***p < 0.001; ****p < 0.0001 indicates a significant difference from the corresponding value of the tumor-free vehicle-treated group by two-way ANOVA followed by Tukey’s multiple comparison test. ^#^p < 0.05 indicates a significant difference from the corresponding value of the tumor-free WFA 2 mg/kg group. ^**α**^p < 0.05 indicates a significant difference from the corresponding value of the tumor-free WFA 4 mg/kg group. ^**¥**^p < 0.05 indicates a significant difference from the corresponding value of the tumor-bearing vehicle-treated group.

While skeletal muscle is primarily thought to be the primary target of cancer-induced cachexia, cardiac muscle is also affected to varying degrees by cancer, resulting in cardiac cachexia [10, 38–40]. To investigate whether ovarian cancer alters the contractile functions of the heart, echocardiography was performed prior to euthanization as described by Bauer et al. [33]. Representative M-mode traces for all the groups are shown in Fig 2A. Echocardiographic assessment revealed that the tumor-bearing vehicle-treated group displayed a significant reduction in heart rate compared to the tumor-free groups (p < 0.0001 for all comparisons) (Fig 2B), likely due to persistent arrhythmias that were detected during the entire duration of the echocardiographic assessment (S2 Fig). These arrhythmias (variable AV blocks) were only detectable in the tumor-bearing vehicle-treated group. Due to experimental limitations, continuous monitoring of heart activity via implantation of a DSI device was not possible due to the fragile health state of this group. Thus, a definitive diagnosis could not be made with respect to the arrhythmias. No significant differences in heart rate within the tumor-free or tumor-bearing WFA-treated groups were observed compared to those of the tumor-free vehicle-treated group (Fig 2B).

As a first step toward assessing LV systolic function, we compared the echocardiographically determined percentage of fractional shortening (FS) (Fig 2B). FS was significantly reduced in the tumor-bearing vehicle-treated group (35.0 ± 3.2% compared to 58.0 ± 2.2% in the tumor-free vehicle-treated group) (p < 0.0001 for all comparisons). The reduction in FS was completely rescued by treatment with WFA at both doses in the tumor-bearing groups compared to that of the tumor-bearing vehicle-treated group (FS: WFA 2 mg/kg = 57.8 ± 3.6%; WFA 4 mg/kg = 55.3 ± 3.2%; p < 0.0001 for both comparisons) (Fig 2B). Similar changes were observed in cardiac output (CO) (Fig 2B), which is heart rate- and stroke volume-dependent [41]. Due to the reduction in heart rate in the tumor-bearing vehicle-treated group, a concomitant decrease in CO was observed (Fig 2B). Along similar lines, because WFA treatment normalized the heart rates of tumor-bearing mice, the CO was similarly normalized (Fig 2B). The trends and levels of significance for the differences in CO mirrored the observed changes in heart rate. WFA treatment did not have any effect on HR or CO in tumor-free mice (Fig 2B).

**Fig 3:**
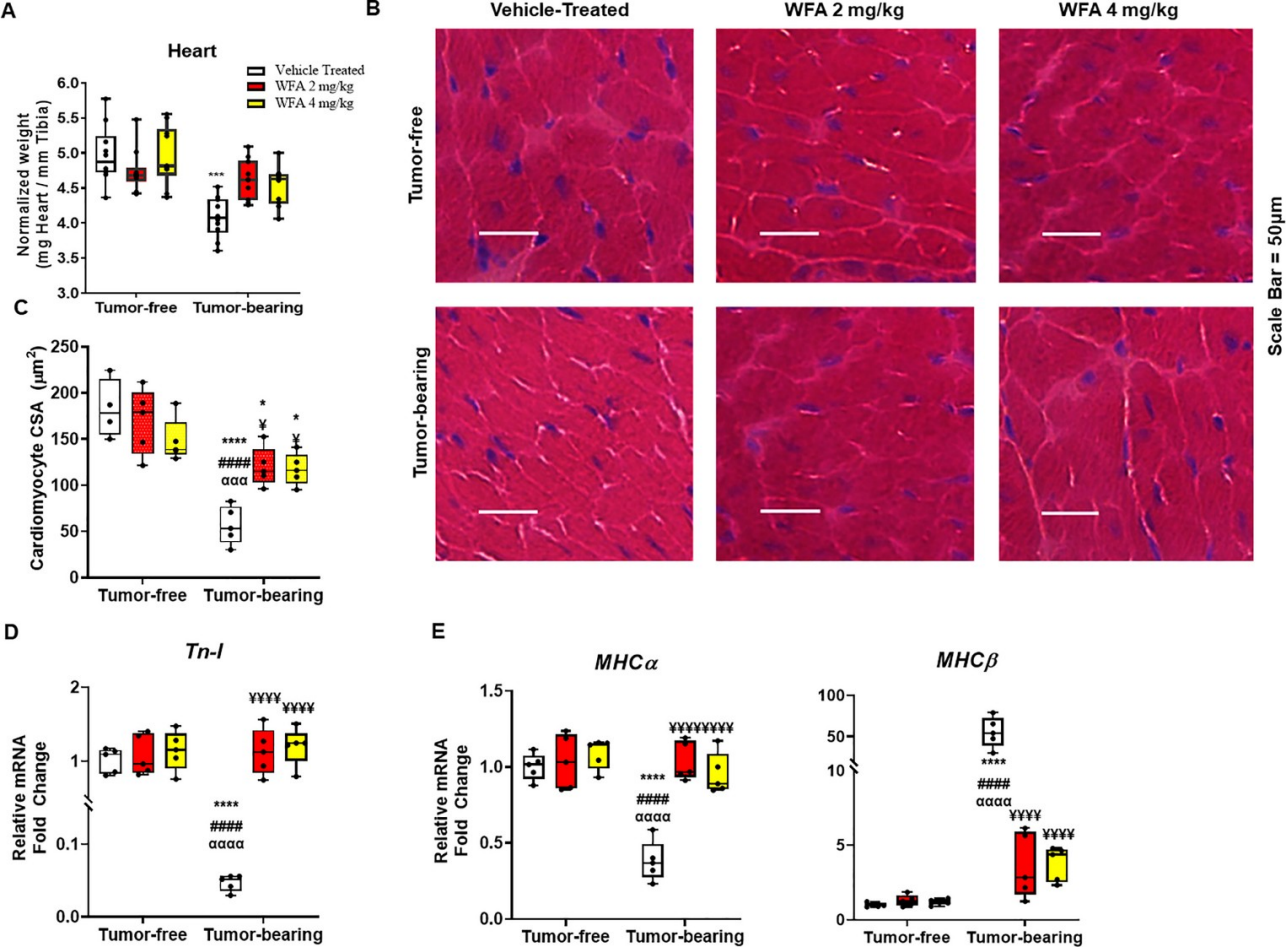
Withaferin A impairs ovarian cancer-induced cardiac atrophy. (**A**) Quantification of heart weight normalized to tibial length in tumor-free and tumor-bearing mice that were treated with WFA or vehicle. (**B**) Representative 40x images of H&E-stained midventricular heart sections. Scale bar  =  50 μm. (**C**) Quantification of average cardiomyocyte CSA. N = 10 per group. Relative mRNA levels of (**D**) *Tn-I*, and (**E**) *MHCα* and *MHCβ*. N = 5 per group. Black circles indicate individual data points. *p < 0.05; **p < 0.01; ***p < 0.001; ****p < 0.0001 indicates a significant difference from the corresponding value of the tumor-free vehicle-treated group by two-way ANOVA followed by Tukey’s multiple comparison test. ^#^p < 0.05 indicates a significant difference from the corresponding value of the tumor-free WFA 2 mg/kg group. ^**α**^p < 0.05 indicates a significant difference from the corresponding value of the tumor-free WFA 4 mg/kg group. ^**¥**^p < 0.05 indicates a significant difference from the corresponding value of the tumor-bearing vehicle-treated group.

One of the potential applications of echocardiography in mice is to noninvasively assess LV mass in experimental animals. We calculated the LV mass of all tumor-free and tumor-bearing animals. No significant difference in LV mass was noted between the tumor-free groups that were treated with vehicle or with either dose of WFA (p > 0.80 for both comparisons) (Fig 2B). However, a significant reduction in LV mass was observed in the tumor-bearing vehicle-treated group (55.6 ± 2.3 mg compared to 91.8 ± 5.5 mg in the tumor-free vehicle-treated group (p < 0.0001 for all comparisons) (Fig 2B). Treatment of tumor-bearing mice with WFA led to a significant increase in LV mass compared to that of the tumor-bearing vehicle-treated group (WFA 2mg/kg: p = 0.0002; WFA 4mg/kg: p < 0.0001). The reduction in LV mass in the tumor-bearing vehicle-treated group could be attributed to cancer-induced atrophy of cardiomyocytes (demonstrated later in Fig 3C). We have not observed any changes in LV mass in tumor-free mice treated with WFA. Previous studies showed cardiac dysfunction and atrophy in cancer models described sex-specific differences in the progression of cardiac cachexia [38]. In a mouse model of colon adenocarcinoma, male mice lost significantly more cardiac mass than females [38]. In our study, we demonstrated a robust cachectic phenotype that more closely resembled the loss of cardiac mass in the male mice of the colon-adenocarcinoma model. To the best of our knowledge, our results clearly indicate that generation of ovarian tumor in mice result in a significant reduction in cardiac function and mass for the first time.

### Withaferin A alleviates ovarian cancer-induced diastolic dysfunction

Some studies have shown that under certain pathological paradigms, systolic function is spared, whereas a dysfunctional state is evidenced in the diastolic phase [42]. Therefore, we next investigated whether ovarian cancer induced diastolic dysfunction. Two common measures of diastolic function, the E/A ratio and the E/e′ ratio, were significantly lower in the tumor-bearing vehicle-treated group (E/A: 0.45 ± 0.12 compared to 2.17 ± 0.05 in the tumor-free vehicle-treated group; E/e’: -18.6 ± 0.6 compared to -9.5 ± 0.65 in the tumor-free vehicle-treated group) than in the tumor-free groups (p < 0.0001 for all comparisons) (Fig 2C). While WFA treatment significantly attenuated systolic dysfunction, diastolic dysfunction was partially improved in the tumor-bearing WFA-treated groups compared to that of the tumor-bearing vehicle-treated group (WFA 2 mg/kg: p = 0.002; WFA 4 mg/kg: p = 0.03), but was still significantly reduced compared to the tumor-free groups (p < 0.0001 for all comparisons) (Fig 2C). Another common diastolic parameter, the isovolumetric relaxation time (IVRT), was significantly prolonged in the tumor-bearing groups compared to those of the tumor-free groups (with no significant differences within the tumor-free or tumor-bearing groups) (Fig 2C). WFA did not affect diastolic function in tumor-free animals. Taken together, these results demonstrate that ovarian cancer induces diastolic dysfunction in the heart (i.e., an impairment in relaxation). Further, while WFA completely preserved systolic function, it did not completely reverse diastolic dysfunction. This suggests that a longer treatment with WFA may be necessary to completely resolve the dysfunctional state of the heart induced by cancer.

### Withaferin A attenuates cardiac cachexia

Morphological changes in the heart, such as atrophy or hypertrophy of cardiomyocytes and collagen deposition/fibrotic scarring are known to cause cardiac dysfunction [43]. Based upon the systolic and diastolic changes associated with our ovarian cancer model and WFA treatment, we investigated changes in the heart at the gross anatomical levels. Xenografting of ovarian cancer cells resulted in a reduction in heart weight (normalized by tibial length) in the tumor-bearing vehicle-treated group compared to tumor-free groups (p < 0.0001 for all comparisons) (Fig 3A). This effect was significantly blunted upon treatment with both doses of WFA in tumor-bearing mice compared to the tumor-bearing vehicle-treated group (p < 0.001 for both comparisons) (Fig 3A). WFA did not have any effect on heart weight in tumor-free animals. We next examined changes in the heart at a morphometric level using H&E staining of mid-ventricular sections of the heart. Similar to the findings in skeletal muscle in our previous study, we observed a significant decrease in the cross-sectional area (CSA) of cardiomyocytes in the tumor-bearing groups compared to those of the tumor-free groups (Fig 3B and 3C). WFA treatment resulted in a partial recovery of the CSA of cardiomyocytes in the tumor-bearing WFA-treated groups compared to those of the tumor-bearing vehicle-treated group (p < 0.0001 for all comparisons) and the tumor-free vehicle-treated group (p = 0.01), but there was no difference between tumor-free animals treated with WFA (Fig 3B and 3C). In addition to changes at the morphometric level, we observed a significant reduction in relative transcript levels of Troponin-I (*Tn-I*; a major contractile protein) in the tumor-bearing vehicle-treated group compared to those of the tumor-free groups (p < 0.0001 for all comparisons) (Fig 3D), This effect was significantly blunted upon treatment with both doses of WFA in tumor-bearing mice compared to the tumor-bearing vehicle-treated group (p < 0.0001 for both comparisons). We also observed a significant shift in MHC isoforms from a predominantly adult α-MHC state to one that is primarily embryonic β-MHC in the tumor-bearing vehicle-treated group compared to the tumor-free vehicle-treated group (Fig 3E). The changes in motor proteins corroborates the functional changes observed in response to the tumor burden.

**Fig 4:**
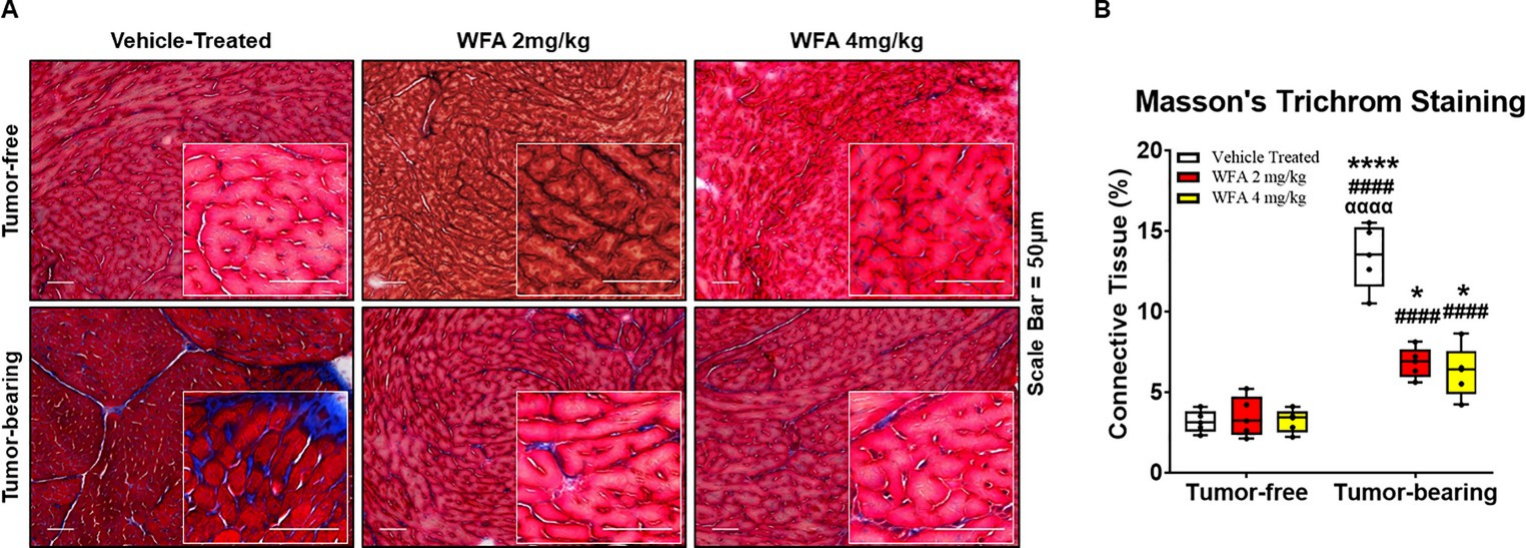
Withaferin A diminishes fibrotic scarring in the heart. (**A**) Representative 20x images of Masson’s trichrome-stained midventricular heart sections in tumor-free and tumor-bearing mice that were treated with WFA or vehicle. Inset images are magnified from the displayed field of view. Scale bar  =  50 μm. (**B**) Quantification of average collagen deposition. N = 10 per group. Black circles indicate individual data points. *p < 0.05; **p < 0.01; ***p < 0.001; or ****p < 0.0001 indicates a significant difference from the corresponding value of the tumor-free vehicle-treated group by two-way ANOVA followed by Tukey’s multiple comparison test. ^#^p < 0.05 indicates a significant difference from the corresponding value of the tumor-free WFA 2 mg/kg group. ^**α**^p < 0.05 indicates a significant difference from the corresponding value of the tumor-free WFA 4 mg/kg group. ^**¥**^p < 0.05 indicates a significant difference from the corresponding value of the tumor-bearing vehicle-treated group.

In addition to atrophy, select models of cancer-induced cachexia result in fibrotic changes in muscle [10]. Therefore, sections from the mid-ventricle of the heart were subjected to Masson’s trichrome staining to elucidate collagen deposition (Fig 4A). A basal degree of blue-stained collagen was clearly noted in the tumor-free vehicle-treated group, but as expected, this collagen deposition was intimately associated with the vasculature (Fig 4A and 4B). A comparable amount of connective tissue was observed in the tumor-free WFA-treated groups compared to that of the tumor-free vehicle-treated group (p > 0.90 for both comparisons). All the tumor-bearing groups exhibited an increase in fibrosis compared to the tumor-free groups (Fig 4B). The percentage of connective tissue was significantly decreased in the tumor-bearing mice that were treated with either dose of WFA compared to the tumor-bearing vehicle-treated group (p < 0.01 for both comparisons), but was significantly higher compared to tumor-free groups (p < 0.001 for all comparisons), suggesting WFA partially rescues the heart from excessive connective tissue formation (Fig 4B). The atrophy and fibrotic changes in the heart are known to result in cardiac dysfunction [44]. Based on heart dysfunction and morphometric changes in cardiac muscle (cardiomyocytes) induced by ovarian cancer, our results clearly demonstrate the induction of cardiac cachexia by ovarian cancer and its reversal by WFA.

### Withaferin A decreases levels of circulating angiotensin II and proinflammatory markers

**Fig 5:**
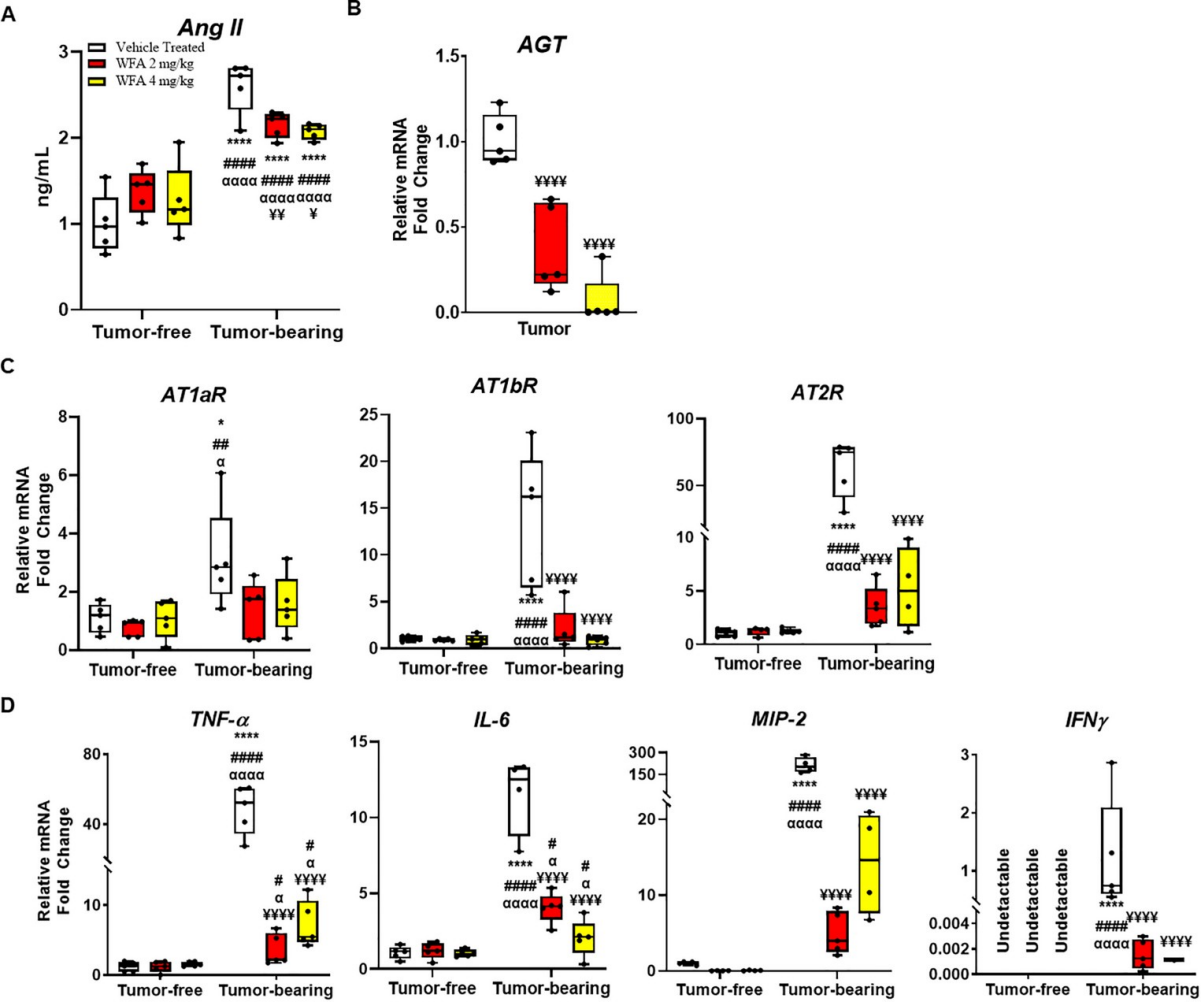
Withaferin A attenuates circulating Ang II levels and pro-inflammatory cytokines in ovarian cancer. (**A**) Levels of Ang II in plasma fractionated from centrally collected blood. (**B**) Relative angiotensinogen mRNA levels in tumor tissues. Relative transcript levels of (**C**) AT_1_R and (**D**) pro-inflammatory cytokines in the heart. N = 5 per group. Black circles indicate individual data points. *p < 0.05; **p < 0.01; ***p < 0.001; or ****p < 0.0001 indicates a significant difference from the corresponding value of the tumor-free vehicle-treated group by two-way ANOVA followed by Tukey’s multiple comparison test. ^#^p < 0.05 indicates a significant difference from the corresponding value of the tumor-free WFA 2 mg/kg group. ^**α**^p < 0.05 indicates a significant difference from the corresponding value of the tumor-free WFA 4 mg/kg group. ^**¥**^p < 0.05 indicates a significant difference from the corresponding value of the tumor-bearing vehicle-treated group.

A few studies have shown that increased levels of circulating Ang II induce myocardial damage, which can lead to cardiac cachexia [45]. Studies have also shown that the increase in circulating levels of Ang II are most likely secreted from cancer cells [46]. To corroborate these findings, we measured Ang II levels in plasma fractionated from centrally collected blood (Fig 5A). Plasma Ang II levels were found to be significantly higher in the tumor-bearing vehicle-treated group compared to the tumor-free vehicle-treated mice (2.7 ± 0.3 ng/ml compared to 1.00 ± 0.34 ng/ml, p < 0.0001) (Fig 5A). WFA treatment significantly reduced the circulating levels of Ang II in the tumor bearing mice compared to the tumor-bearing vehicle-treated group (WFA 2 mg/kg: 2.15 ± 0.015 ng/ml, p < 0.01; WFA 4 mg/kg: 2.07 ± 0.08 ng/ml, p < 0.01) (Fig 5A). In addition, we measured the relative transcript levels of angiotensinogen in tumors from the tumor-bearing mice. Treatment with WFA significantly reduced relative transcript levels of angiotensinogen in tumor samples collected from mice (WFA 2 mg/kg: 0.3676 ± 0.252 fold change; WFA 4 mg/kg: 0.0694 ± 0.144 fold change compared to 1.0081 ± 0.144 fold change in the vehicle-treated group, p < 0.0001) (Fig 5B).

It has been reported that ovarian cancer prognosis, tumor angiogenesis, and patient outcome are correlated with angiotensin II receptor 1 and 2 levels (AT_1_R, AT_2_R) [18]. Humans have one isoform of AT_1_R, whereas mice have two subtypes, AT_1a_R and AT_1b_R [47]. We measured relative transcript levels of *AT*_*1a*_*R*, *AT*_*1b*_*R*, and AT_2_R in the heart in all of the tumor-free and tumor-bearing groups. We observed a significant increase in *AT1aR* transcription in cardiac tissues of tumor-bearing vehicle-treated mice (3.14 ± 1.7 fold change compared to tumor-free vehicle-treated mice, p < 0.01) (Fig 5C), as well as for *AT*_*1b*_*R* (13.86 ± 7.2 fold change, p < 0.0001) (Fig 5C). We observed a 62.89 ± 21.13 (p < 0.0001) fold increase of AT_2_R in tumor-bearing animals compared to control, which was significantly reduced in WFA treated tumor-bearing animals (WFA 2 mg/kg: 3.5 ± 1.8, WFA 4 mg/kg: 5.2 ± 3.2) (Fig 5C).

The mechanisms of Ang II-induced muscle atrophy are complex and include potential direct effects of inflammatory cytokines such as IL-6, TNF-α, and IFN-γ [48]. Therefore, we assessed the expression of NF-κB-related pro-inflammatory cytokines in the heart myocytes (Fig 5D). As shown in Fig 5D, the relative transcript levels of all proinflammatory cytokines assessed (*TNFα*, IL-6, *MIP-2* (the mouse ortholog of human IL-8), and *IFNγ*), were significantly increased in the tumor-bearing vehicle-treated group and were significantly reduced in the WFA-treated groups compared to the tumor-free vehicle-treated group (*p* < 0.0001) (Fig 5D). The relative mRNA levels of *IFNγ* were found to be undetectable in tumor-free animals, so this component is normalized to the tumor-bearing vehicle-treated group to allow a partial analysis (Fig 5D). The transcript levels of *IFNγ* were detectable in the tumor-bearing vehicle-treated mice and were significantly reduced in the WFA-treated groups (Fig 5D).

## Discussion

Involuntary weight loss and a reduction in skeletal muscle mass remain the focus of cachexia research. Recent studies have also provided evidence that cancer-induced cachexia also causes significant cardiac atrophy, resulting in a state of cardiac cachexia and contributing to chronic heart failure [49]. However, there is no information if ovarian cancer induces cardiac cachexia and to date no effective drug is available to treat it. Objective of the present study was to establish the cardiac cachexia phenotype, and to investigate the possibility of WFA against cancer-induced cardiac cachexia.

A review of the current literature suggests that the most common models of cancer-induced cachexia are the C26 colon cancer, Lewis lung carcinoma, and Apc^min/+^ models. Interestingly, some of these models of cancer-induced cachexia demonstrate varying degrees of concomitant cardiac atrophy [10, 50, 51]. A significant reduction in heart weight and cardiac dysfunction were observed in male mice; however, this was significantly diminished in female mice [10, 50, 51]. Indeed, most studies on cachexia exclude the utilization of female lab animals, likely due to the more prevalent phenotype exhibited in males. As such, there is a fundamental lack of knowledge about not only the skeletal muscle effects, but also the cardiac effects of cancer-induced cachexia in female lab animals.

Indeed, most studies of cachexia focus on the skeletal muscle aspect without consideration of cardiac muscle [2, 52–57]. In the current study, we characterized the cachectic effects associated with *in vivo* growth of A2780 ovarian cancer xenografts at a functional level with respect to both cardiac and skeletal muscle (unpublished data). Consistent with our previous study [21], we observed gross body changes (Fig 1) and effects on skeletal muscle atrophy associated with tumor burden confirming our results for the development of a cachectic phenotype [21], suggesting that female lab animals can in fact be utilized to study/model for both skeletal muscle and cardiac cachexia. Extensive studies have addressed the mechanisms underlying the atrophying effects of cancer on skeletal muscle, but the effects on cardiac muscle remain less understood.

With respect to skeletal muscle, cachexia causes hypercatabolism of sarcomeric motor proteins [55]. This impairment in skeletal muscle contraction facilitates atrophy. Cancer induces a shift in metabolism to a predominantly glycolytic state [58]. Skeletal muscle myofibers in turn undergo oxidative to glycolytic myofiber-type conversions [58]. This is mediated by upregulation of embryonic myosin heavy chain (MHC) proteins and downregulation of adult MHCs [38, 58, 59]. Interestingly, cardiac failure is also characterized by switching gene expression from adult to fetal MHC isoforms [38, 59], potentially serving as surrogate markers for the induction of cardiac cachexia. MHCα, which is predominant in adult mouse hearts, has higher ATPase activity than MHCβ, which is predominant during embryonic development [59]. Even a small magnitude of isoform shift could significantly impact heart function [38, 59]. Our qPCR results demonstrated a significant reduction in relative transcript levels of *MHCα* and a significant increase in *MHCβ* in the tumor-bearing groups (Fig 3), which might be an adaptive response of the heart to conserve energy that contributes to the damaged contractile function in tumor-bearing mice. Additionally, cardiac troponin-T and troponin-I are regulatory proteins that control the calcium-mediated interaction between myosin and actin [59, 60]. Previous studies have shown that elevated levels of troponin-T are contingent upon the death of cardiomyocytes [59]. Studies evaluating cardiac cachexia have not shown much of an effect on troponin-T. However, in the context of cardiac cachexia, troponin-I (a key myofilament protein) is altered in response to changes in the contractility of the heart, as shown in Fig 3. In our studies, we, demonstrated tumor-induced cardiac remodeling and myocardial dysfunction (Fig 2) which are consistent with other studies focused on C26 colon cancer in males [61, 62].

Many studies have demonstrated effect of WFA on apoptosis of cardiomyocytes [63]. Our previous work have shown that WFA attenuated skeletal muscle cachexia [21]. In current study we show microscopic analysis of the hearts of tumor-bearing mice showed enhanced intermyofibrillar collagen deposition and cardiomyocyte atrophy (Figs 3 and 4). Indeed, a significant degree of fibrosis was evidenced in the heart in the tumor-bearing vehicle treated group. Approximately 20% of the area within the heart was deeply stained for collagen, similar to what has been observed in colon cancer models of cachexia [10, 38, 59]. Fibrosis of cardiac tissue commonly occurs in myocardial pathologies and is known to cause a decrease in the performance of the heart. This collagen deposition causes stiffening of the heart, which in turn leads to worsening of heart failure, primarily via diastolic dysfunction. Interestingly, due to an absence of noticeable symptoms, diastolic dysfunction is relatively underdiagnosed compared to systolic dysfunction [64]. In our study, we observed both systolic and diastolic dysfunction in the hearts of tumor-bearing mice. Promisingly, WFA preserved systolic function and partially improved diastolic function, which could be attributed to the decrease in fibrous scarring in the heart [44].

We have reported in our previous study that WFA has an anti-inflammatory ability by direct inhibition of NF-κB signaling on skeletal muscle [21], based on this data we hypothesized that WFA could ameliorate the cachectic phenotype exhibited in ovarian cancer. Tumor-induced cardiac remodeling involves increased levels of pro-inflammatory cytokines, such as IL-1β, IL-6, and IL-8, as well as ventricular thinning and decreased troponin-I levels, as shown in Figs 2 and 5, resulting in dysfunctional contraction and relaxation of the heart (Fig 2). The concomitant induction of skeletal muscle and cardiac muscle cachectic phenotypes suggest the presence of a similar mechanism of induction. Nevertheless, WFA treatment promisingly attenuated or completely rescued the effects induced by the xenografted ovarian cancer.

TNFα is a pro-inflammatory cytokine with a wide range of biological effects that has been implicated in the pathophysiology of many cardiovascular diseases [65–67]. TNFα is central in initiating and sustaining the pro-inflammatory cytokine cascade, and simulates the production of other cytokines, such as IL-6 and IL-8 [65, 68]. TNFα induces myocardial fibrosis by inhibiting phagocytosis of collagen in the heart [65, 69]. Our qPCR data demonstrated a drastic increase in *TNFα* transcription with a concomitant increase in fibrosis in tumor-bearing mice, which was reduced by WFA treatment. WFA is known to inhibit activation of the NF-κB pathway, resulting in a downregulation of pro-inflammatory cytokines, such as TNFα and IL-6 [21, 70, 71] [21, 71]. TNFα, IL-6, IL-8, IL-1β, and IFNγ are considered to be the major inflammatory mediators of cancer-induced cachexia, which have been demonstrated to be elevated in various animal models of cancer [62, 66, 69]. Our qPCR data suggest that local inflammation was increased in the hearts of tumor-bearing mice, as demonstrated by the increase in relative transcript levels of *IL-6* and *MIP-2* transcription. TNFα contributes to Ang II-induced adverse cardiac remodeling [65]. Ang II importantly contributes to the induction of skeletal muscle wasting, which is partially mediated by AT_1_R. In skeletal muscle, it was demonstrated that an AT_1_R inhibitor was sufficient in attenuating the induction of a cachectic phenotype [62]. Studies have shown that overexpression/activation of AT_2_R attenuated ischemia-induced cardiac remodeling [72, 73]. However, controversial findings are reported recently. Overexpressing AT2R specifically in ventricular cardiomyocytes decreased cardiac contractility and dilated cardiomyopathy, while severity of cardiac dysfunction was positively correlated with level of AT_2_R expression [74, 75]. Xu et al [76] showed that the level of overexpression determines beneficial or detrimental role of AT_2_R to the heart: low or moderate levels of overexpression of AT_2_R protects the heart against ischemia-induced injury, meanwhile this protection is inversely related to the overexpression levels of AT2R [76]. In addition to the effect on skeletal muscle, our data suggests AT_1_R and AT_2_R inhibition could be beneficial for cardiac muscle.

## Conclusion

**Fig 6:**
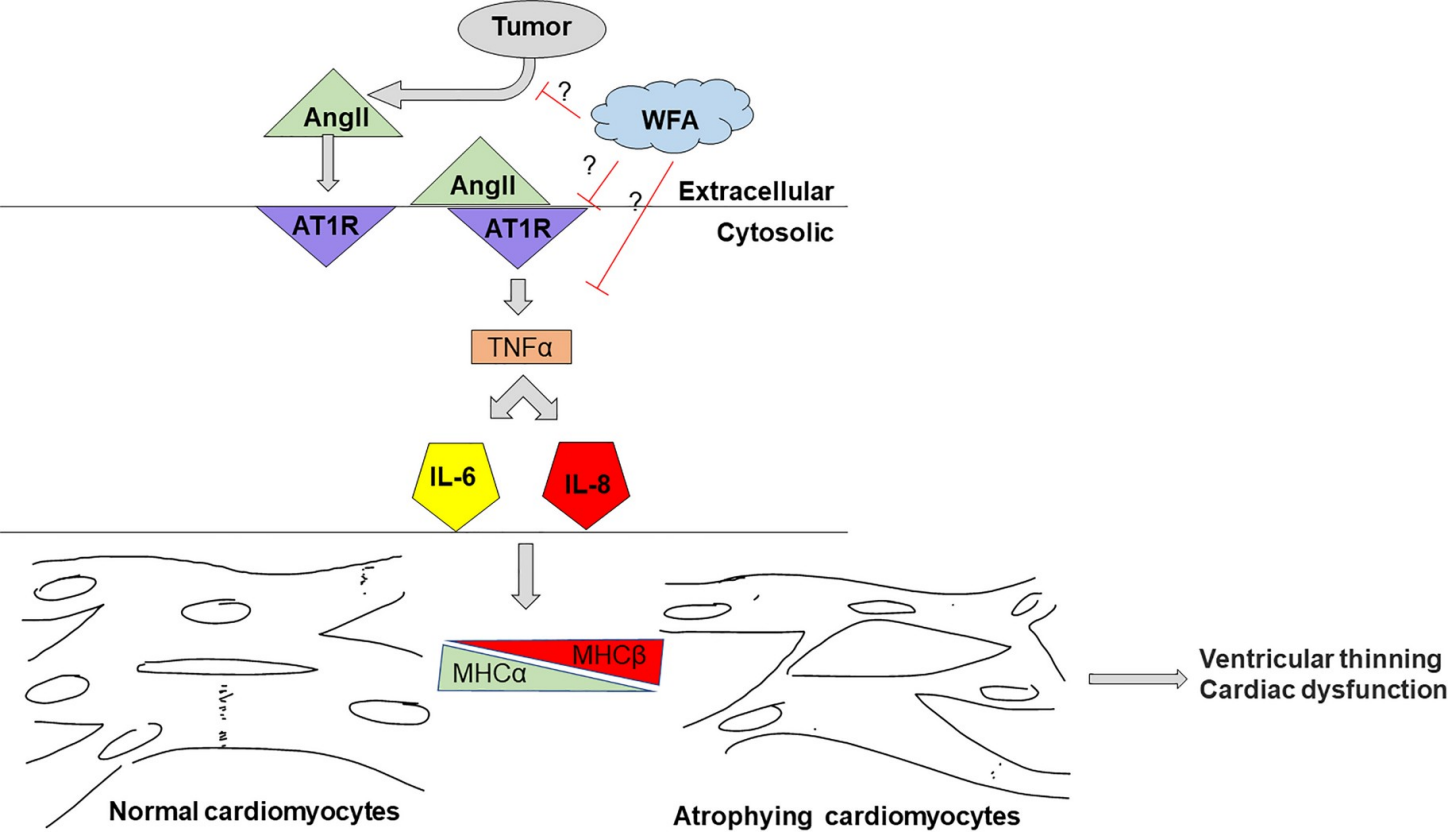
Schematic representation of our hypothesis. we hypothesize that xenografting of ovarian cancer into female mice would induce a cachectic phenotype in cardiac muscle through AT1R. Ang II released from tumor induces shift in MHC isoforms from a predominantly adult α-MHC state to one that is primarily embryonic β-MHC in the tumor-bearing vehicle-treated group compared to the tumor-free vehicle-treated group and caused cardiac cachexia. Treatment with WFA would reduce Ang II levels and attenuates cardiac cachexic phenotype.

Our xenograft model of ovarian cancer suggests that, given the proper oncological paradigm, female lab animals can and in fact should be utilized in studies of cachexia. The results of our study underscore the intersection between cancer and the heart. The major findings of this study are as follows: (i) We observed a cardiac cachectic phenotype in our model. (ii) This cardiac cachectic phenotype was rescued by treatment with WFA, (iii) WFA treatment attenuates plasma Ang II levels in tumor-bearing mice, and (iv) Pro-inflammatory markers could have been induced through AT_1_R in tumor-bearing mice, and these increases were abrogated by WFA treatment (Fig 6). However, it remains unknown whether WFA reverses heart dysfunction in established cardiac cachexia after 1 week of xenografting or if WFA prevents the development of cardiac cachexia.

## Supporting information

S1 Fig Metastatic localization of ovarian cancer in female immunodeficient mice. (**A**) Representative images of the abdominal/peritoneal cavity in vehicle-treated tumor-free and tumor-bearing mice showing the replacement of fat pads with metastatic tumor lesions. (**B**) Representative images of the spleens of vehicle-treated tumor-free and tumor-bearing mice showing organ encapsulation/replacement of the adherent perisplenic fat pad.
(PPTX)

S2 Fig Tumor predisposes mice to developing arrhythmias. Representative M-mode recordings of parasternal long axis views of the hearts of (**A**) tumor-free vehicle-treated, (**B**) tumor-bearing vehicle-treated, (**C**) tumor-bearing WFA 2 mg/kg-treated, and (**D**) tumor-bearing WFA 4 mg/kg-treated mice.
(PPTX)

S1 Table Organ weights in tumor-free and tumor-bearing mice. Organs are weight and normalized on initial body weight.
(PPTX)
