# Supplementary material for: Withaferin A attenuates ovarian cancer-induced cardiac cachexia
Source: PLoS One. 2020 Jul 28;15(7):e0236680. doi: 10.1371/journal.pone.0236680 (PMC7386592; doi:10.1371/journal.pone.0236680)
Supplement: S1 Fig — (A) Representative images of the abdominal/peritoneal cavity in vehicle-treated tumor-free and tumor-bearing mice showing the replacement of fat pads with metastatic tumor lesions. (B) Representative images of the spleens of vehicle-treated tumor-free and tumor-bearing mice showing organ encapsulation/replacement of the adherent perisplenic fat pad. (PPTX) [file pone.0236680.s001.pptx]

## Slide 1
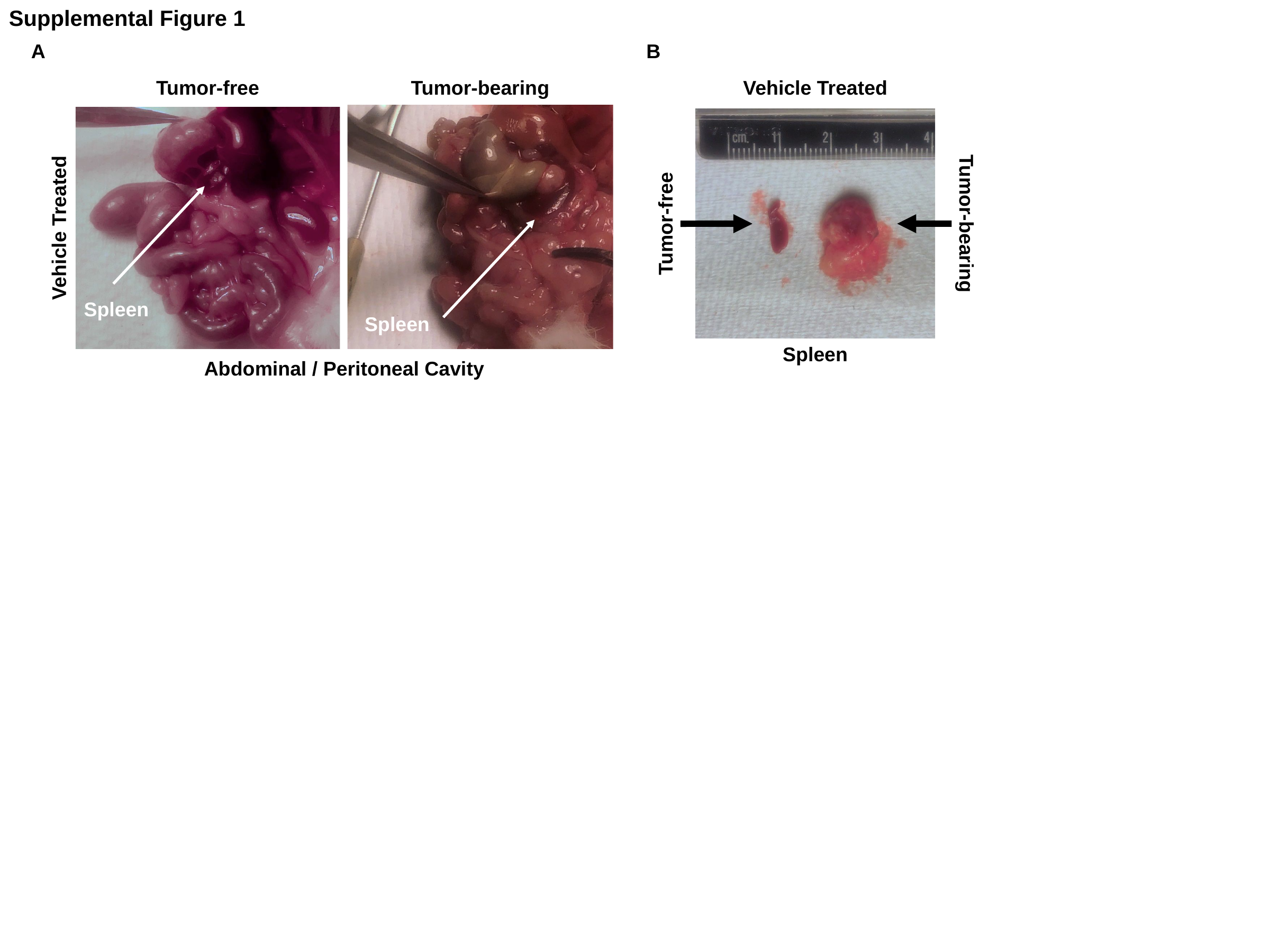

Supplemental Figure 1
A
B
Tumor-free
Tumor-bearing
Vehicle Treated
Spleen
Spleen
Abdominal / Peritoneal Cavity
Vehicle Treated
Tumor-free
Tumor-bearing
Spleen
