# Supplementary material for: Withaferin A attenuates ovarian cancer-induced cardiac cachexia
Source: PLoS One. 2020 Jul 28;15(7):e0236680. doi: 10.1371/journal.pone.0236680 (PMC7386592; doi:10.1371/journal.pone.0236680)
Supplement: S2 Fig — Representative M-mode recordings of parasternal long axis views of the hearts of (A) tumor-free vehicle-treated, (B) tumor-bearing vehicle-treated, (C) tumor-bearing WFA 2 mg/kg-treated, and (D) tumor-bearing WFA 4 mg/kg-treated mice. (PPTX) [file pone.0236680.s002.pptx]

## Slide 1
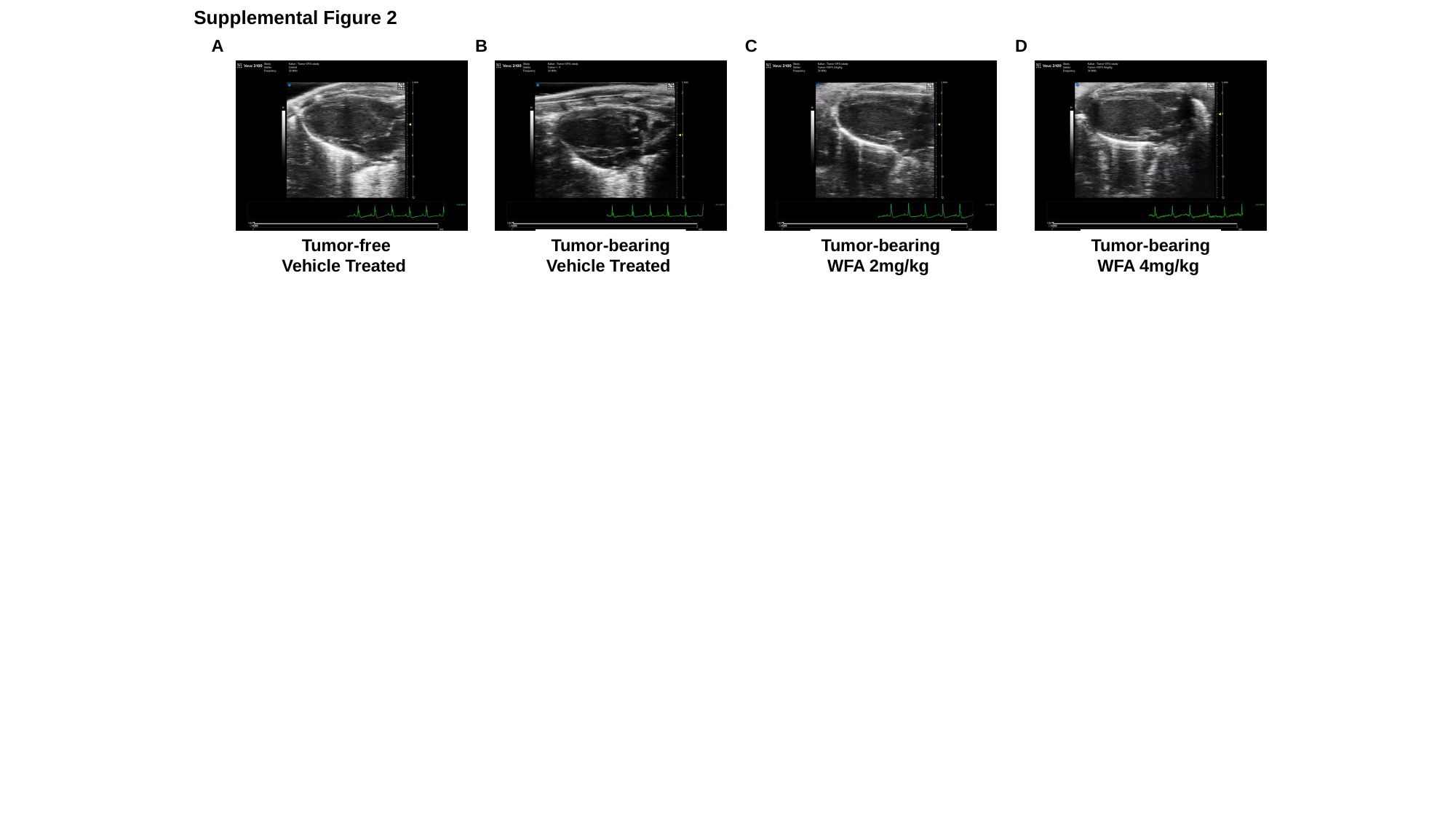

Supplemental Figure 2
A
B
C
D
Tumor-free
Vehicle Treated
Tumor-bearing
Vehicle Treated
Tumor-bearing
WFA 2mg/kg
Tumor-bearing
WFA 4mg/kg
