# Supplementary material for: Withaferin A attenuates ovarian cancer-induced cardiac cachexia
Source: PLoS One. 2020 Jul 28;15(7):e0236680. doi: 10.1371/journal.pone.0236680 (PMC7386592; doi:10.1371/journal.pone.0236680)
Supplement: S1 Table — Organs are weight and normalized on initial body weight. (PPTX) [file pone.0236680.s003.pptx]

## Slide 1
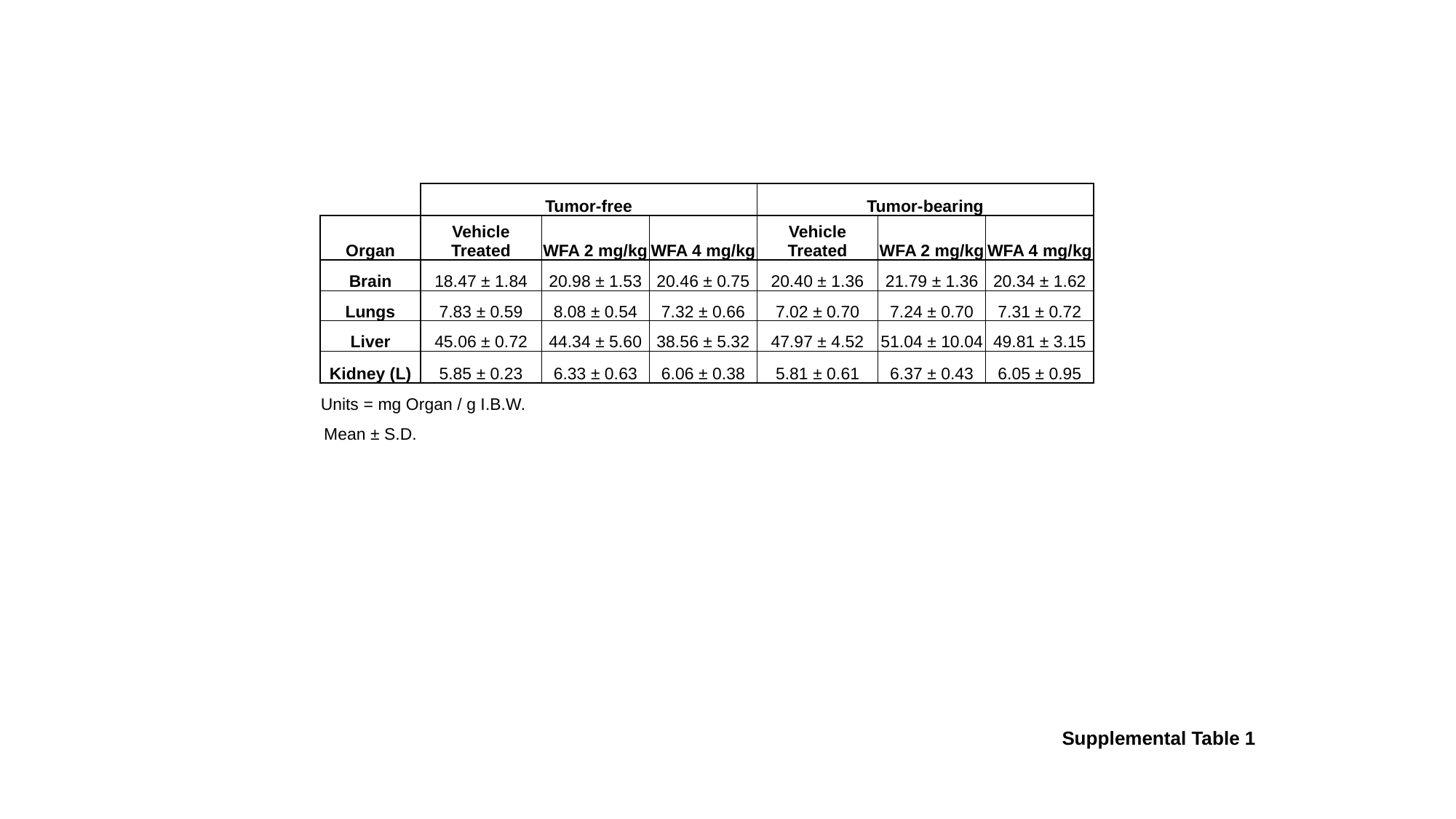

| | Tumor-free | | | Tumor-bearing | | |
| --- | --- | --- | --- | --- | --- | --- |
| Organ | Vehicle Treated | WFA 2 mg/kg | WFA 4 mg/kg | Vehicle Treated | WFA 2 mg/kg | WFA 4 mg/kg |
| Brain | 18.47 ± 1.84 | 20.98 ± 1.53 | 20.46 ± 0.75 | 20.40 ± 1.36 | 21.79 ± 1.36 | 20.34 ± 1.62 |
| Lungs | 7.83 ± 0.59 | 8.08 ± 0.54 | 7.32 ± 0.66 | 7.02 ± 0.70 | 7.24 ± 0.70 | 7.31 ± 0.72 |
| Liver | 45.06 ± 0.72 | 44.34 ± 5.60 | 38.56 ± 5.32 | 47.97 ± 4.52 | 51.04 ± 10.04 | 49.81 ± 3.15 |
| Kidney (L) | 5.85 ± 0.23 | 6.33 ± 0.63 | 6.06 ± 0.38 | 5.81 ± 0.61 | 6.37 ± 0.43 | 6.05 ± 0.95 |
| Units = mg Organ / g I.B.W. | | | | | | |
| Mean ± S.D. | | | | | | |
| | | | | | | |
Supplemental Table 1
